# A Cross-Sectional Study Analyzing Recent Trends in the Treatment of Acne Vulgaris: Prescription Patterns and Demographic Data From the 2018-2019 National Ambulatory Medical Care Survey (NAMCS) Database

**DOI:** 10.7759/cureus.79170

**Published:** 2025-02-17

**Authors:** Arash Pour Mohammad, Gordon H Bae

**Affiliations:** 1 Department of Dermatology, Iran University of Medical Sciences, Tehran, IRN; 2 Department of Dermatology, Stanford University School of Medicine, Palo Alto, USA

**Keywords:** acne vulgaris, isotretinoin, national ambulatory medical care survey, spironolactone, treatment trends

## Abstract

Introduction: Acne vulgaris is one of the most common inflammatory skin conditions worldwide, with notable psychosocial ramifications. Despite the availability of multiple treatment options, evolving clinical guidelines and healthcare policies may influence prescription practices over time. This study examines the latest trends in acne management using recent data from the National Ambulatory Medical Care Survey (NAMCS).

Methods: We analyzed 2018-2019 NAMCS data to characterize acne treatment patterns across all age groups in outpatient settings in the United States. Treatments encompassed oral isotretinoin; hormonal therapies, such as oral contraceptive pills (OCPs) and spironolactone; systemic antibiotics, including minocycline, doxycycline, sarecycline, and tetracycline; and topical therapies, such as benzoyl peroxide, topical antibiotics, topical retinoids, azelaic acid, and salicylic acid.

Results: A total of 8,756,594 acne-related visits were recorded, with 41.5% occurring among patients aged 13-19 years. Female patients constituted 65.4% of visits. Isotretinoin emerged as the most frequently prescribed treatment among women (31.1%), followed by oral antibiotics (17.4%) and spironolactone (11.2%). Spironolactone usage nearly doubled compared to prior data (5.1%-11.2%), while oral antibiotic prescriptions decreased from 22.9% to 17.4%. The use of OCPs slightly declined (5.7%-4.5%).

Conclusion: Our findings indicate a shift in acne management patterns, characterized by a rise in antiandrogenic therapies and a reduction in oral antibiotic use, suggesting heightened antibiotic stewardship efforts. The elevated frequency of isotretinoin prescriptions likely reflects the iPledge program requirements for periodic follow-up visits rather than an actual increase in disease severity. While these trends underscore ongoing changes in clinical practice, further longitudinal research is warranted to assess how these prescription shifts influence long-term patient outcomes.

## Introduction

Acne vulgaris is a common inflammatory skin condition affecting 9.4% of the global population, particularly prevalent among adolescents and young adults aged 16-20 years, with peak rates reaching 47% [1,2]. Acne poses psychological and social challenges, emphasizing the need for effective treatment strategies [3].

The clinical management of acne has evolved significantly, reflecting advancements in understanding its pathophysiology and the development of novel therapeutic modalities. Despite these advancements, oral antibiotics remain a cornerstone for moderate-to-severe cases due to their proven efficacy and tolerability. However, their nonselective and prolonged use has led to an emerging public health threat of antibiotic resistance [4,5]. Recent findings indicate that nearly 20% of patients have been continuously prescribed oral antibiotics for more than six months, significantly exceeding the recommended three to four months [6].

This increasing deviation from guidelines underscores the necessity of regularly analyzing treatment patterns to ensure that evolving practices align with the latest clinical evidence [7]. This study updates current acne treatment trends using the latest data from the National Ambulatory Medical Care Survey (NAMCS).

## Materials and methods

Data source

We analyzed data from the 2018-2019 NAMCS to assess trends in acne treatment across diverse age groups. NAMCS, a nationally representative survey by the National Center for Health Statistics, captures outpatient medical services in the United States. The survey collects comprehensive, anonymized data encompassing diagnostic codes, treatment modalities, patient demographics, and healthcare settings [8].

We selected this period to update findings from previous data (2002-2016), as the 2018-2019 data are the most recent publicly available. Data for the intervening year of 2017, as well as subsequent years (2020-2023), have not yet been released publicly [9]. Detailed descriptions of the survey's methodology are available on the NAMCS website [8].

Study population and tools

Our study population included all patients diagnosed with acne, identified using the International Classification of Diseases, 10th Revision codes L70.0 (acne vulgaris) and L70.9 (acne, unspecified). The population was stratified into the following age groups: <13, 13-19, 20-29, 30-39, 40-49, and 50 years and older. Demographic information, including age, race, ethnicity, and insurance status, was extracted. The treatments analyzed were categorized according to the American Academy of Dermatology's 2016 guidelines for treating acne, chosen to align with our study period [10].

The oral treatment category included isotretinoin, hormonal agents, such as FDA-approved oral contraceptive pills (OCPs) and spironolactone, and systemic antibiotics, such as minocycline, tetracycline, doxycycline, and sarecycline. The topical treatment category included benzoyl peroxide (BPO), topical antibiotics (clindamycin, erythromycin, and dapsone), combination of BPO/topical antibiotics (erythromycin/BPO, clindamycin/BPO), topical retinoids (tretinoin, adapalene, and tazarotene), combination of topical retinoids with BPO/topical antibiotics (adapalene/BPO and clindamycin/tretinoin), azelaic acid, and salicylic acid. Patients who received multiple treatments from different categories, whether prescribed individually or as part of a combination agent, were systematically classified under the respective combination treatment category. Medications from the same drug class were combined and counted once for each visit. Table 1 includes the relevant medication codes used.

**Table 1:** Codes and names of extracted oral and topical acne medications

Medications	Code
Oral medications	
Isotretinoin	d01245
Drospirenone-ethinyl estradiol	d04760
Norgestimate-ethinyl estradiol	d03781
Ethinyl estradiol-norethindrone	d03238
Drospirenone, ethinyl estradiol, levomefolate calcium	d07697
Spironolactone	d00373
Minocycline	d00110
Tetracycline	d00041
Doxycycline	d00037
Sarecycline	d09003
Sulfamethoxazole-trimethoprim	d00124
Trimethoprim	d00123
Topical medications	
Benzoyl peroxide topical	d01246
Erythromycin topical	d03200
Clindamycin topical	d01241
Dapsone topical	d05543
Minocycline topical	d04761
Benzoyl peroxide-erythromycin	d03857
Benzoyl peroxide-clindamycin	d04742
Tretinoin	d01244
Adapalene	d04015
Tazarotene	d04138
Clindamycin-tretinoin	d05973
Adapalene-benzoyl peroxide	d07382
Azelaic acid topical	d03848
Salicylic acid topical	d01307
Topical acne drugs (unspecified)	c00143

Ethical considerations

The study utilized deidentified, publicly available data from the NAMCS. All research activities were conducted in compliance with ethical standards for human research.

Statistical analyses

Patient visit weights were applied to the data to provide estimates representative of the national population. There was no sampling as we analyzed the entire relevant dataset to ensure comprehensive coverage and accuracy in depicting national treatment patterns for acne vulgaris. Descriptive statistics, such as medians and interquartile ranges for continuous variables and percentages for categorical variables, were used. Independent sample t-tests were performed to compare the proportions of treatments prescribed in the current and previous periods. A p value of less than 0.05 was considered statistically significant. All statistical analyses were conducted using Statistical Package for the Social Sciences version 26 (IBM Corp., Armonk, NY).

## Results

A total of 8,756,594 weighted visits with acne diagnoses were recorded, of which 41.5% involved patients aged 13-19 years. Women accounted for 65.4% of cases (F/M ratio 1.89). The majority of 58.4% were non-Hispanic White, and 85.6% of treatments were covered by private insurance (Table 2).

**Table 2:** Demographic information of patients diagnosed with acne ^*^Visit weights have been applied to represent national estimates ^†^Data unavailable in the dataset CHIP: Children's Health Insurance Program

Parameters		Weighted count (median)*	Interquartile range (%)
Median age (years)		26	16-33
Age category	<13	445,657	5.1%
	13-19	3,633,010	41.5%
	20-29	2,215,293	25.3%
	30-39	873,305	10.0%
	40-49	758,418	8.7%
	>50	830,910	9.5%
	Total	8,756,594	100.0%
Gender	Female	5,730,580	65.4%
	Male	3,026,014	34.6%
Ethnicity	Hispanic or Latino	2,463,509	28.1%
	Non-Hispanic or Latino	6,293,084	71.9%
Race	White	7,536,309	86.1%
	Black	600,026	6.9%
	Other	620,259	7.1%
Race/ethnicity	Non-Hispanic White	5,113,545	58.4%
	Non-Hispanic Black	589,272	6.7%
	Hispanic	2,463,509	28.1%
	Non-Hispanic other	590,268	6.7%
Type of payment	Unknown^†^	42,379	0.5%
	Private insurance	7,463,636	85.6%
	Medicare	56,992	0.7%
	Medicaid, CHIP, or other state-based program	614,541	7%
	Self-pay	514,851	5.9%
	Other	28,229	0.3%

Among female patients, isotretinoin was the most commonly prescribed treatment, accounting for 31.1% of visits, followed by oral antibiotics at 17.4% and spironolactone at 11.2%. OCPs were prescribed to 4.5% of female patients, while retinoids combined with BPO or topical antibiotics were prescribed in 11.3% of visits. Topical antibiotics alone were utilized in 10.8% of treatments, and the combination of BPO plus topical antibiotics was seen in 3.5% of cases. Age-related trends revealed that isotretinoin was most frequently prescribed to female patients aged 20-29 (68.0%), while spironolactone was prominent in the 30-39 age group (29.8%). Oral antibiotics were notably prescribed to female patients over 50 (57.3%). Azelaic acid was rarely used only in female patients over 50 (3.4%). Salicylic acid was not reported in any group, and unspecified acne drugs were noted in 2.1% of all female patients (Table 3).

**Table 3:** Distribution of different medication categories prescribed by age group and gender ^*^Patients receiving retinoids and not receiving any other medication categories such as topical or oral antibiotics, spironolactone, OCPs, retinoid, or BPO compounds ^†^All patients receiving any topical antibiotic OCPs: oral contraceptive pills; BPO: benzoyl peroxide

Gender	Age categories	Weighted no. of all patients	Isotretinoin	Oral antibiotics (minocycline, tetracycline, doxycycline, sarecycline)	Spironolactone	OCPs	Retinoids only^*^	Retinoids + BPO/topical antibiotics	BPO + topical antibiotics	Topical antibiotics^†^
Female	<13	408,644	3.3%	0%	0%	0%	2.9%	6.5%	0%	18.1%
	13-19	1,468,768	11.4%	17.5%	10.5%	6.4%	25%	26.5%	11.7%	23.4%
	20-29	1,884,367	68%	9.4%	5.2%	6.6%	1.4%	7.5%	1.5%	2.9%
	30-39	714,003	7.1%	5.7%	29.8%	4.2%	13.4%	0%	0%	11.0%
	40-49	513,650	48.3%	19.3%	29.9%	2.3%	4.1%	11%	0%	7.2%
	>50	741,148	3.0%	57.3%	3.6%	0%	24.6%	4.3%	0%	4.5%
	Total	5,730,580	31.1%	17.4%	11.2%	4.5%	12.3%	11.3%	3.5%	10.8%
Male	<13	37,013	0%	0%	0%	0%	0%	0%	68.7%	0%
	13-19	2,164,243	12.1%	12.1%	3.8%	0%	19.1%	10%	0%	2.1%
	20-29	330,927	40.3%	17.1%	0.0%	11.4%	9.8%	14.6%	7.7%	16.5%
	30-39	159,302	14.7%	65.9%	0%	0%	0%	0%	0%	0%
	40-49	244,768	77.2%	0%	0%	0%	4.7%	0%	2.9%	0%
	>50	89,761	26.1%	0%	0%	0%	0%	0%	0%	0%
	Total	3,026,014	20.8%	14%	2.7%	1.3%	15.1%	8.8%	1.9%	3.3%
All patients		8,756,594	27.6%	16.2%	8.3%	3.4%	13.3%	10.4%	2.9%	8.2%

During 2018-2019, 33% of female patient visits involved isotretinoin prescriptions. This notable frequency may partly reflect the iPledge program's requirements in the United States, which necessitate monthly visits for patients on isotretinoin due to its teratogenic potential. These requirements contribute to the observed increase in prescription frequency, as patients are compelled to return regularly for monitoring and prescription renewals. This is significant since isotretinoin prescription rates from the earlier period, 2002-2016, were not available, making comparisons challenging.

There was a noticeable rise in the prescription of spironolactone, increasing from 5.1% in the earlier period to 11.2%, suggesting an increasing preference for antiandrogenic agents in the treatment of acne (t = 465.528, df = 5,730,579, p < 0.001; mean difference = 6.143%, 95% CI = 6.12-6.17). Conversely, the use of oral antibiotics decreased from 22.9% to 17.4%, which may reflect heightened awareness and efforts toward antibiotic stewardship (t = -346.745, df = 5,730,579, p < 0.001; mean difference = -5.492%, 95% CI: -5.52 to -5.46). OCPs slightly decreased from 5.7% to 4.5% (t = -135.183, df = 5,730,579, p < 0.001; mean difference = -1.174%, 95% CI: -1.19 to -1.16). However, OCPs were still administered in 6.4% and 6.6% of female patients aged 13-19 and 20-29, respectively.

## Discussion

The findings identify crucial trends in acne vulgaris management, marked by significant shifts in prescription practices possibly driven by healthcare policies and patient demographics. A notable observation is the inflated prescription rate of isotretinoin in 33% of female patient visits, which appears high not solely due to clinical preference but also because of the iPledge program's stringent requirements for safe, monitored use due to its teratogenic risks. This program mandates regular follow-up visits, potentially inflating prescription statistics and reflecting regulatory compliance rather than an actual increase in acne severity or prevalence. Additionally, there was an increase in spironolactone use from 5.1% to 11.2%, indicating a shift toward antiandrogenic agents and a decline in oral antibiotic use from 22.9% to 17.4%, likely due to enhanced antibiotic stewardship efforts. Although there was a slight decrease in OCP usage from 5.7% to 4.5%, their use remains relatively stable among younger female patients.

A study by Perche et al. discussed the impact of the 2009 Global Alliance to Improve Outcomes in Acne guidelines, emphasizing antibiotic stewardship through a recommended three-month limit on oral antibiotic use and the promotion of topical retinoids for maintenance therapy [7,11]. Around the same period, multiple reports documented a substantial 36.6% drop in dermatologist-prescribed antibiotics between 2008 and 2016 and a marked 391% rise in spironolactone prescriptions [12,13]. In parallel with these broader trends, our data show a notable decrease in oral antibiotic use (from 22.9% to 17.4%) and a twofold increase in spironolactone prescriptions (from 5.1% to 11.2%), underscoring the growing preference for antibiotic-sparing strategies in acne management.

The FDA’s iPledge program monitors isotretinoin use by categorizing patients as either unable to become pregnant or able to become pregnant. Those in the latter group must practice abstinence or use two approved contraceptive methods if sexually active [14]. Although iPledge appears to have decreased the overall number of isotretinoin prescriptions among individuals of childbearing potential, there remain gaps in patient education regarding the most effective contraceptive options, such as intrauterine devices or subdermal implants, compared to oral contraceptive pills [14]. Additionally, the program’s binary categorization has drawn criticism for limiting access to isotretinoin for transgender male patients, who may experience acne flares linked to testosterone therapy [15].

The strength of this study lies in its utilization of the comprehensive NAMCS database, ensuring our analysis reflects a nationally representative view of current outpatient acne treatment practices in the United States. We enhance the precision of our findings by excluding patients with multiple diagnoses, allowing for a more accurate assessment of acne treatment patterns. Furthermore, by comparing our results with prior studies, we identify key trends such as the increased use of antiandrogenic treatments and the reduced reliance on oral antibiotics. Our adherence to the American Academy of Dermatology’s 2016 guidelines ensures that our analysis is aligned with current clinical standards, providing a critical evaluation of their real-world application. Additionally, the recency of our data (2018-2019) offers a timely update on prescribing trends, filling a gap in the literature and ensuring that our insights are relevant and useful for informing contemporary dermatological practice and policy.

A key limitation of this study is the potential underreporting of treatments, as over-the-counter medications like BPO and adapalene may not be captured without a formal prescription. Additionally, the lack of detailed data for systemic agents such as oral antibiotics and OCPs, which might be prescribed for conditions unrelated to acne, complicates the interpretation of their true role in acne management. Moreover, the prescription of spironolactone and OCPs to a small proportion of male patients (2.7% and 1.3%, respectively) could reflect use in individuals with self-identified gender differences, such as those undergoing hormone therapy. However, the dataset's lack of explicit identifiers for gender identity limits precise interpretation. Another significant limitation arises from the cross-sectional design of the NAMCS dataset, which records visits rather than unique patients, preventing tracking of individual treatment progress or outcomes and possibly duplicating data entries for patients making multiple visits.

## Conclusions

This study provides a comprehensive analysis of acne treatment patterns using the most recent NAMCS data. Our findings indicate a marked shift toward the use of antiandrogenic agents such as spironolactone and a decreasing reliance on oral antibiotics, reflecting ongoing efforts in antibiotic stewardship. While the usage of isotretinoin appears inflated due to iPledge program mandatory monitoring requirements, caution is warranted in interpreting these trends as definitive indicators of clinical practice evolution.

Furthermore, these shifts highlight a broader responsiveness within dermatology to evolving clinical guidelines and public health initiatives aimed at reducing antibiotic resistance. The growing reliance on antiandrogenic agents signals an increased focus on addressing hormone-driven pathophysiology as a sustainable alternative to prolonged antibiotic use. Future studies incorporating patient outcomes and provider-level variables will be critical to understanding how these trends impact long-term treatment efficacy, patient satisfaction, and the prevalence of antibiotic resistance. These insights will help refine evidence-based acne management strategies and support the continued evolution of dermatologic care.
